# Combined Methodologies for Determining *In Vitro* Bioavailability of Drugs and Prediction of *In Vivo* Bioequivalence From Pharmaceutical Oral Formulations

**DOI:** 10.3389/fchem.2021.741876

**Published:** 2021-11-03

**Authors:** A. De Simone, L. Davani, S. Montanari, V. Tumiatti, S. Avanessian, F. Testi, V. Andrisano

**Affiliations:** ^1^ Department of Drug Science and Technology, University of Turin, Torino, Italy; ^2^ Department for Life Quality Studies, University of Bologna, Rimini, Italy; ^3^ Valpharma International S.p.A., Rimini, Italy

**Keywords:** levonorgestrel, bioavailability, drug–excipient interaction, gastrointestinal passive permeability, PAMPA, bioequivalence, dissolution model

## Abstract

With the aim of developing an *in vitro* model for the bioavailability (BA) prediction of drugs, we focused on the study of levonorgestrel (LVN) released by 1.5 mg generic and brand-name tablets. The developed method consisted in combining a standard dissolution test with an optimized parallel artificial membrane permeability assay (PAMPA) to gain insights into both drug release and gastrointestinal absorption. Interestingly, the obtained results revealed that the tablet standard dissolution test, combined with an optimized PAMPA, highlighted a significant decrease in the release (15 ± 0.01 μg min^−1^ vs 30 ± 0.01 μg min^−1^) and absorption (19 ± 7 × 10^–6^ ± 7 cm/s Pe vs 41 ± 15 × 10^–6^ cm/s Pe) profiles of a generic LVN tablet when compared to the brand-name formulation, explaining unbalanced *in vivo* bioequivalence (BE)*.* By using this new approach, we could determine the actual LVN drug concentration dissolved in the medium, which theoretically can permeate the gastrointestinal (GI) barrier. In fact, insoluble LVN/excipient aggregates were found in the dissolution media giving rise to non-superimposable dissolution profiles between generic and brand-name LVN tablets. Hence, the results obtained by combining the dissolution test and PAMPA method provided important insights confirming that the combined methods can be useful in revealing crucial issues in the prediction of *in vivo* BE of drugs.

## Introduction

The development of an *in vitro* method for the prediction of drugs’ BA is an interesting challenge, mostly because of the possibility to save both time and money in the approval process of a given drug. The prediction of drugs’ BA *in vitro* can be especially useful in those studies aimed at determining the BE of a generic and a brand-name drug. Indeed, despite the public concern in having access to generic drugs as quickly as possible due to their lower price, the regulatory process for their approval takes time like any other process related to this aspect (Chazin et al., 2020). However, if the chemical equivalence between two chemical entities is relatively easy to establish, it is more complex to prove the BE between two or more formulations, with the same active pharmaceutical ingredients (APIs). The differences noticed in BE for the studied products can be mainly related to the physical properties of the excipients used in the compared formulations (Zarmpi et al., 2017). The ability of detecting the factors influencing the physicochemical properties, as well as drug delivery, will make it possible to thoroughly depict both its BA and BE profiles (Niazi, 2007).

Most of the studies comparing a generic drug to a brand-name product, in terms of BE, include the determination of active compounds or their metabolites in biological fluids. Other studies may involve comparative pharmacodynamic (PD) investigations in humans or comparative clinical trials. Nevertheless, it is not always possible or necessary to use *in vivo* human data for evaluating drug bioavailability, at least in the formulation optimization process (Chen et al., 2001a). A large number of physicochemical and physiologic factors that may influence the adsorption of drugs can indeed be monitored by applying some designed *in silico* (Mathias and Crison, 2012) and *in vitro* models. For all these reasons, the application of model-based drug development, as well as precise and accurate analytical methods supported by statistical considerations (Lalonde et al., 2007; Kim et al., 2018; Martinez and Zhao, 2018; Zhao et al., 2019), seems to offer a reliable approach for the *in vitro* characterization of a drug before *in vivo* BE studies. For these reasons, there is a great need to develop surrogate models able to provide information about the BA or BE profile for all the classes of studied drugs. Starting from the fact that the BA of a drug, released from a solid oral form, is influenced by dissolution, solubility, and intestinal permeability, the proposed methods should provide the characterization of both the dissolution and adsorption profiles. This is pivotal considering the need for pharmaceutical industries to develop and apply methods able to evaluate the bioavailability of a large fraction of poorly water-soluble drug compounds (Ku and Dulin, 2012; Sironi et al., 2017). Indeed, many *in vitro* models have been developed to evaluate the capacity of new drug delivery systems or formulations to enhance the permeability of poorly soluble drugs (Buyukozturk et al., 2010; Kanzer et al., 2010; Fischer et al., 2011a; Fischer et al., 2011b; Fischer et al., 2012). Despite the meaningful results obtained by these types of experiments, it is also essential to consider that, before any absorption can take place, the drug needs to be in solution. Indeed, such drugs can often exhibit an unreal high absorption rate if the experimental setup does not take into account the dissolution of the drug prior to membrane permeation. For this purpose, some combined dissolution/permeability models have been proposed (Ginski et al., 1999; Kobayashi et al., 2001; Kataoka et al., 2003; Kataoka et al., 2006; Motz et al., 2007; Kataoka et al., 2011; Kataoka et al., 2014). Some of the proposed models mimicking the *in vivo* environment were developed in order to forecast the oral absorption of pH-independent and -dependent drugs in a more and more reliable manner (Kobayashi et al., 2001; He et al., 2003; Kataoka et al., 2003; Noureddine et al., 2005; Sugawara et al., 2005; Kataoka et al., 2006). The application of these systems able to assess both the dissolution and the permeation process was found to be particularly suitable for poorly soluble drugs. However, their application still remains very limited in the conventional drug discovery process because of the various drawbacks shown. In particular, for low aqueous solubility molecules, the precipitation of drugs in the aqueous buffer system may represent a critical step. At the same time, sufficient concentrations of the solubilized drug in both the donor and the receiver compartment must be guaranteed to carry out a permeability assay and ensure measurement sensitivity.

With all this in mind, the aim of this work was to validate an *in vitro* system combining dissolution and PAMPA for the assessment of both the dissolution and the *in vitro* oral adsorption profile of LVN from oral formulations. The designed method, combining both the tests, was intended to provide an integrated approach for BE prediction of the drug before moving on *in vivo* studies.

The dissolution test is routinely used for stability and quality control studies. The reliability of this kind of assay in predicting the *in vivo* performance of a drug product is related to its capability of reconstructing *in vitro* the distinctive conditions registered in the gastrointestinal (GI) tract (Dressman et al., 1998). Indeed, the FDA has now recognized the major role of this assay in reducing the regulatory burden in predicting the human studies in generic drug development (Anand et al., 2011). Moreover, according to the biopharmaceutics classification system (BCS) approach, which classifies drug substances into four primary groups (highly soluble/highly permeable, highly permeable/poorly soluble, highly soluble/poorly permeable, and poorly soluble/poorly permeable), the highly permeable/highly soluble drug substance formulated into a rapidly dissolving drug product may need only *in vitro* dissolution studies to establish its BA (Amidon et al., 1995; Dahan et al., 2009). As for the permeability (Pe) test, several methods such as *in situ* rat intestinal perfusion (Kim et al., 2006), the *ex vivo* rat intestinal tissue, the MDCK (Irvine et al., 1999) and Caco-2 (Artursson, 1990) cell monolayers, and the PAMPA (Avdeef, 2005) have been optimized to simulate drugs’ absorption across biologic membranes, and it can be applied for permeability determination. These models are mostly suitable for the description of permeability across the GI membrane. Moreover, different cellular and non-cellular models combined with dissolution systems have been developed and adapted in order to evaluate the Pe even of poorly soluble drug entities (Buckley et al., 2012). With the intention to give mechanistic insights or evaluate the effect of different excipients on the performance of formulations, many approaches combining dissolution and Caco-2 permeation testing were suggested (Ginski et al., 1999; Kataoka et al., 2003; Kataoka et al., 2006; Motz et al., 2007). Since the application of the Caco-2 model unfortunately presents many drawbacks, more innovative approaches, employing non-cellular biomimetic barriers, were developed for the same purpose (Fischer et al., 2011b; Fischer et al., 2012; Gantzsch et al., 2014). Other methods affected the development of dialysis-based dissolution/permeation models (Lovering and Black, 1973; Blanquet et al., 2004). Unfortunately, even these methods are partially flawed, mainly due to the interaction of amphiphilic weak basic and acidic drugs with such biomimetic membranes (Bibi et al., 2016). None of the suggested models specifically concerns the prediction of BE for two oral formulations containing the same API. Since the experimental setup, in this case as well, should predict the *in vivo* behavior of tested formulations, we combined the dissolution test previously described to the PAMPA one to achieve this objective. The PAMPA, using the phospholipid artificial membrane, is considered a suitable model for the detection of passive transport of epithelial cells. Due to its versatility, this method is more suitable for the detection of passive permeability than the Caco-2 model, even for poorly soluble drugs (Buckley et al., 2012). Moreover, it offers several features such as low cost and higher reproducibility that make it certainly more suitable for the high-throughput setup as a first approach than cellular models. The PAMPA model is now widely used for the assessment of passive transport of epithelial cells (Avdeef et al., 2007). Indeed, despite the real-life properties and reliable outcomes provided by cellular models applied to permeability studies (Borchardt et al., 2011), the PAMPA method is to be preferred for the determination of passive permeability, since it overcomes such issues as long cultivation time, high cost, high degree of variability and low capacity in characterizing poorly soluble drugs (Buckley et al., 2012; Berben et al., 2018). Indeed, the PAMPA model is described by Handbook of Bioequivalence Testing as a cost-effective and high-throughput method (Niazi, 2007). Moreover, due to its ability of providing the benefits of a more biologically relevant system, it is considered a very common assay nowadays (Avdeef, 2005).

The combination of the dissolution test and PAMPA method was applied to characterize and compare the BA of LVN released from a generic formulation (formulation A) to that of LVN released from the brand-name tablets (formulation B). This was done because *in vivo* BE failed after performing only a dissolution study. The idea is based on the awareness that the combination of the dissolution test with the PAMPA gives the opportunity to characterize the studied drugs in terms of solubility and absorption and to eventually point out some peculiarities that might influence these parameters in the studied formulations. The application of UV spectroscopy for the determination of LVN released and absorbed during the proposed assays was found to be more predictive of *in vivo* studies, since it guarantees the direct quantification of the real amount of analyte. Indeed, the direct UV analysis turned out to be appropriate for these studies, avoiding further modifications in the sample dissolution that might compromise the results, which may occur by using a reversed-phase HPLC method with the organic modifier.

Hence, starting from the necessity of understanding the different *in vivo* BE profiles shown by the two LVN oral formulations, the developed method was found to be able to provide a new set of information and recommendations useful for resolving existing or emerging problems in BE studies of new oral formulations, more capable of predicting the *in vivo* behavior.

## Materials and Methods

### Materials

HPLC-grade acetonitrile and ethanol (VWR, Radnor, Pennsylvania, United States) and water obtained from the Milli-RX apparatus (Millipore, Burlington, Massachusetts, United States) were used to prepare solutions and mobile phases. Sodium dodecyl sulfate (SDS) was purchased from Panreac Quimica (Barcelona, Spain). 37% hydrochloric acid (HCl) was obtained from VWR (Radnor, Pennsylvania, United States).

The water solution for dissolution studies was prepared by mixing 0.1% SDS in 0.1N HCl solution. The solutions were filtered through a 0.45 μm membrane filter and degassed before their use in HPLC.

### UV Spectrophotometry

The spectrophotometric analyses were performed on a Jasco V-530 double beam spectrophotometer, using a 1 cm quartz cell. Suitable settings were a slide width of 2 nm, scan speed of 400 nm min^−1^, and UV range of 210–450 nm. A stock solution was prepared by dissolving the appropriate amount of LVN in ethanol in order to obtain 1 mg/ml solution. LVN analytical solutions were obtained by diluting to volume with 0.1% SDS 0.1N HCl solution (0.37–6 μg/ml).

#### Calibration Graph

The zero-order UV spectra of LVN (0.375–6 μg/ml in 0.1% SDS 0.1N HCl) were recorded using 0.1% SDS 0.1N HCl solution as the blank; the absorbance values at *λ*_max_ = 245 nm were plotted against the corresponding concentration to obtain the calibration graph.

### HPLC Analysis

The HPLC Waters Alliance apparatus comprised a Waters Alliance 2489 UV detector, a Waters Alliance e2695 separation module, and a Waters column heater (Waters, Milford, MA, United States). The chromatographic separations were performed on a 2.5 μm C8 Luna (150 mm × 4.6 mm i.d.) column (Phenomenex, Torrance, CA, United States) kept at 30°C, using a mobile phase consisting of acetonitrile: water at 60:40 (v/v) at a flow rate of 1.0 ml/min. UV detection at 247 nm was used.

#### Reference Solution

A 1.5 μg/ml LVN solution was obtained by transferring about 30 mg of LVN working standard, exactly weighted, in a 200 ml volumetric flask. The powder was dissolved by sonication in 150 ml of ethanol. The solution was cooled down and brought to volume with the same solvent. 2 ml of the obtained solution was diluted to 200 ml with dissolution medium (0.1N HCl containing 0.1% SDS) in order to obtain 1.5 μg/ml solution.

#### Calibration Graph

A stock solution of LVN (0.15 mg/ml) was prepared in the mobile phase of acetonitrile:water at 55:45 (v/v). This solution was then used to prepare standard solutions of LVN (0.3–3.0 μg/ml) by diluting to volume with 0.1N hydrochloric acid containing 0.1% sodium dodecyl sulfate. Each standard solution was injected in triplicate into the chromatograph; the peak areas were plotted against the corresponding LVN concentrations to obtain the calibration graph.

#### Samples Analysis

At least seven injections of the dissolution medium or as many as needed to obtain a good baseline were performed. Then, the standard solution was injected five times. The standard deviation of the areas was verified to be less than 2.0%. Then, the six dissolution samples derived from the dissolution test were analyzed.

### Dissolution Test

In order to determine the LVN kinetics of release from formulations A and B in a time-course experiment, a dissolution experiment was carried out following the procedure reported in European Pharmacopoeia 9.0 (2.9.3. Dissolution Test for Solid Dosage Form). Apparatus 2 (paddle) described in European Pharmacopoeia 9.0 and a thermostatic bath regulated at 37 ± 0.5°C were used. The apparatus consisted of six vessels, each one equipped with a paddle. Three vessels for each tablet type were used. 0.1N hydrochloric acid containing 0.1% sodium dodecyl sulfate was adopted as the dissolution medium. The experiments were carried out under the following conditions: 1,000 ml dissolution medium volume, the rotation speed of the paddle of 75 ± 3 rpm, and the distance of the plate from the bottom of the vessel of 25 ± 2 mm. An equal volume of dissolution medium was poured in each of the six glass vessels. The liquid was kept at 37 ± 0.5°C. Every single tablet obtained by two different manufacturers was transferred in the vessel before starting the test. 3 ml of solution was withdrawn from each vessel from an intermediate zone between the surface of the dissolution medium and the top of the paddle, and not less than 1 cm from the vessel wall. The solutions were then filtered through a 0.45 µm polypropylene (PP) filter and subjected to determination of LVN using the chromatographic conditions described above.

For the spectroscopic determination of LVN released during the dissolution test, the samples obtained as previously described were subjected to centrifugation at 1,500 rpm for 3 min. The collected samples were later analyzed by both RP-HPLC, as described in *HPLC Analysis*, and UV spectrophotometry (*UV Spectrophotometry*) in the wavelength range from 210 to 450 nm.

### Dissolution Test in 100 ml Volumetric Flask

Both formulations A and B were left under constant stirring in 0.1N hydrochloric acid (Sigma-Aldrich) containing 0.1% sodium dodecyl sulfate (Sigma-Aldrich) solution at 37°C.

An aliquot of 1 ml of solution was withdrawn at different times from each flask. The UV spectra of the collected samples were registered after centrifugation at 1,500 rpm for 3 min in the wavelength range from 210 to 450 nm.

### PAMPA Test Validation

The quality control compounds atenolol, carbamazepine, coumarin, norfloxacin, and ranitidine hydrochloride (Sigma-Aldrich) of known intestinal permeability were used to validate the analysis set. Stock solutions of the reference drugs were prepared in DMSO (Sigma-Aldrich) at 10 mm and then diluted to reach the concentration of 500 μM in PBS of pH = 7.4, so that the concentration of DMSO does not exceed 5% of the total volume. The acceptor 96-well microplate (MultiScreen®, catalog no. MASSACCEPTOR from Millipore) was filled with 180 μl of pH = 7.4 PBS solution containing 5% of DMSO. The donor 96-well plate (MultiScreen® IP Sterile Plate PVDF membrane, pore size of 0.45 µm, catalog no. MAIPN4510 from Millipore) was coated with 5 μl fresh solution of L-α-phosphatidylcholine from egg yolk (Sigma-Aldrich) in dodecane (20 mg ml^−1^) and left at 70°C for 5 min.

Then, it was filled with drug solution (180 ul per well). The obtained “sandwich” was left under constant slight shaking (50 rpm) overnight at 30°C.

### LVN Stability Test

In order to investigate the stability of LVN in PAMPA conditions, the absorbance of the analyte was registered at different concentrations ranging from 6 to 30 μg/ml in 0.1% SDS 0.1N HCl. The parameters were set as reported in *UV Spectrophotometry*. The stability was monitored over 6 days.

### PAMPA Test Applied to 1.5 mg LVN Tablets

An exact aliquot of 50 ml of the solutions obtained from 35′ and 75′ dissolution tests, performed in 100 ml flasks, was transferred to a Falcon tube and centrifuged for 10 min at 1,400 rpm at RT. Then, 10 ml of supernatant for each sample was transferred to a 15 ml Falcon tube and centrifuged under the described conditions. The acceptor 96-well microplate was filled with 180 μl of 0.1% SDS 0.1N HCl solution. The donor 96-well plate was prepared as previously described. Then, the wells were filled with 180 μl of drug solution (at least four wells for each sample). The “sandwich” was left for 1, 1.5, 2, and 4 h at 37°C under continuous slight shaking (50 rpm). After incubation, the solutions in the acceptor plate wells were collected for each sample, and the UV spectra were registered. A Jasco V-630Bio spectrophotometer (Jasco, Tokyo, Japan) was used for the UV measurements.

## Results and Discussion

This work was conceived after the failure of LVN *in vivo* bioequivalence studies, which were planned after only dissolution studies were carried out on the two formulations. In-house HPLC dissolution studies of generic and brand-name formulations did not show any differences in the LVN kinetics and equilibrium parameters. Therefore, the two formulations were considered “similar” and submitted to *in vivo* studies. However, the application of *in vitro* models for the evaluation of excipient effects on API solubility and permeability is crucial in particular for drugs which exhibit poor aqueous solubility (Ku and Dulin, 2012). Indeed, especially for these kinds of compounds, the demand for fast and economical *in vitro* models able to appraise the effects of new formulations on drugs’ BA is urgent. Since even BE may be affected by drug/excipient interactions, providing insights into dissolution and permeability profiles becomes crucial to predict and certify the BE of generic formulations compared to the corresponding brand-name ones. Moreover, the ability of predicting BA and BE profiles of a new drug gives the opportunity to avoid generic formulations which behave differently from the brand-name ones.

Both the definitions of BA and BE imply the use of pharmacokinetic measures to evaluate the rate of the drug released from the medicinal product and the amount of drug absorbed into the systemic circulation (Chen et al., 2001a). Hence, whatever the different approaches adopted in establishing the BE or BA profile of a drug are, the most important goal is to assess the rate and extent of drug absorption (el-Tahtawy et al., 1998; Chen et al., 2001b; Tozer et al., 1996; el-Tahtawy et al., 1994), since the obtained information will make it possible to consider the safety and the effectiveness of the studied drugs. So, the application of *in vitro* methodologies, able to predict the *in vivo* drug release and absorption from the solid oral formulation, has to be considered the key point to predict both the BA and the BE of oral products (Leslie, 1993).

All things considered, in order to offer new insights into a specific case, we developed a new approach whose scope may be extended to every oral formulation. Indeed, the new method was designed to be applied to the early detection of important formulation implications that might affect LVN BE *in vivo*.

**FIGURE 1:**
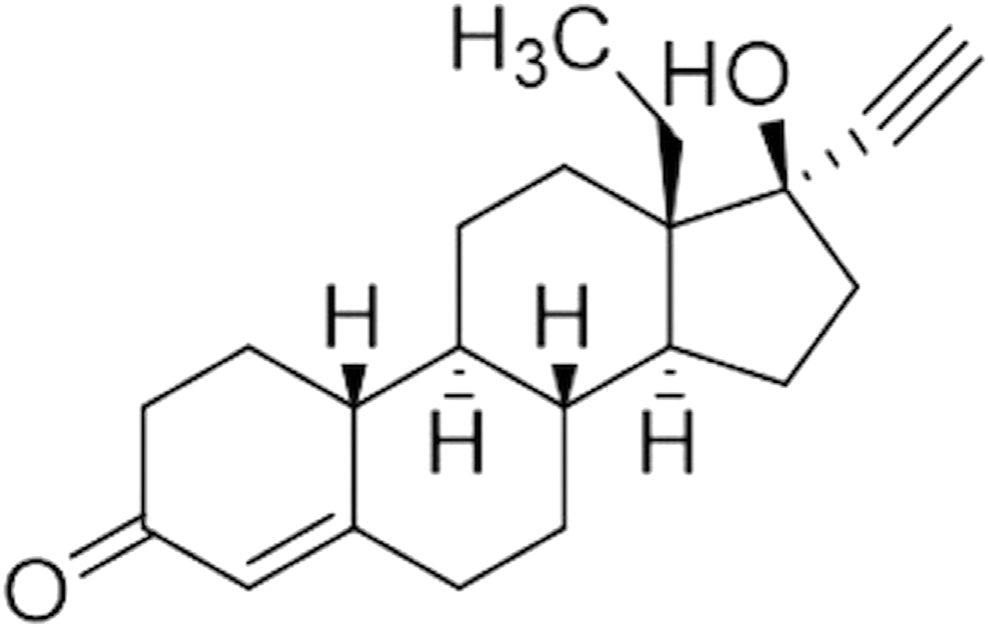
LVN structure.

In this regard, we focused on the optimization and development of a combined analytical approach for the *in vitro* characterization of dissolution and absorption profiles of LVN released by 1.5 mg generic and brand-name tablets. LVN is one of the most widely used progestogens (Figure 1), and different dosage forms containing this API are on the market. The formulations we studied are classified as emergency contraceptive pills (ECPs) (Kitanova, 2019). According to the BCS (Amidon et al., 1995), LVN belongs to class I since it is considered a highly soluble [Guidance for industry, 2000; Multisource (generic) pharmaceutical products, 2000; Committee for Medicinal Products for Human Use, 2008] and highly permeable drug (Lindenberg et al., 2004). Indeed, it is characterized by a BA of almost 100%. This parameter is not influenced by a first-pass effect of the liver. LVN is rapidly and completely absorbed after oral administration. Indeed, with a single 1.5 mg tablet, a *C*_max_ of 20 ng/ml with a *T*_max_ of 1.4 h has been observed (Devoto et al., 2005).

In order to detect the reasons for the failure of the *in vivo* BE studies (data not shown) of these formulations, we combined a dissolution test following the criteria proposed by the European Pharmacopoeia and a PAMPA study to predict permeability. Indeed, once the rate of LVN time-course release by the dissolution test was established by UV spectroscopy, in comparison with the HPLC assay, the kinetics of permeation was measured by the PAMPA test, under optimized conditions, to identify any differences in LVN dissolution and absorption profiles. Indeed, what matters most is that the reliability of all these measurements regarding the release, along with the rate and extent of absorption of an active pharmaceutical ingredient (API), makes them applicable to studies intended to assure comparable therapeutic effects among different formulations.

### Dissolution Test

The *in vivo* dissolution process is the first step on which the curative effect of a drug depends. The pharmaceutical products, in solid oral dosage forms, indeed have to undergo dissolution in the GI fluid, before being absorbed and reaching the systematic circulation. The results obtained from the dissolution test, in the field of BE studies, might be influenced by the physicochemical status of drugs and excipients and by the differences in applied manufacturing processes (Paus et al., 2015). In light of this, the dissolution assay can significantly reduce the number of *in vivo* studies, but it is also useful to assess batch-to-batch quality and support batch release, to provide process control and quality assurance and to assess the need for further BE studies. As mentioned before, in the case of such molecules as LVN, endowed with high solubility and absorption rate, the application of the dissolution test can be considered adequate for BA determination.

In the present study, the dissolution test was carried out for both 1.5 mg LVN formulations A and B, following the procedure reported by European Pharmacopoeia 9.0 (2.9.3. Dissolution Test for Solid Dosage Form. Apparatus 2) (Europe, 2016). The used apparatus consisted of six vessels equipped with a paddle which represents the stirring element. An equal number of three vessels were designed for each tablet type. The medium used for the dissolution test was 0.1N hydrochloric acid containing 0.1% sodium dodecyl sulfate (SDS). In order to obtain an LVN kinetics release plot from the dissolution test, HPLC and UV analyses were carried out.

#### HPLC Analysis

Different solutions were withdrawn from paddles at 15, 30, 45, and 60 min. The solutions containing LVN released by formulation A and formulation B were then analyzed by HPLC coupled with the UV detector (Görög, 2011). The method was validated by determining linearity, reproducibility, and specificity. Specificity was assessed by analysis under the same chromatographic conditions and the LVN and blank formulations, verifying no interferences eluting at the LVN retention time. The linearity of the method was assessed by a calibration curve obtained using standard solutions of LVN at different concentrations ranging from 0.30 to 3.00 μg/ml. The obtained equation was *y* = 74,149.7× + 75.8 (*R* ^2^ = 1.000). The samples withdrawn from paddles were replaced with an equal volume of dissolution media and injected in the HPLC system after being filtered. The obtained peak area for each sample was then compared to the standard average area obtained injecting five times a 1.5 μg/ml LVN standard solution (theoretical concentration). The percentage of the active ingredient released was calculated by the following formula:

$$\%=\frac{\mathrm{SAMPLE}\;\mathrm{AREA}\times \mathrm{Ws}\times \mathrm{Wu}}{\mathrm{STANDARD}\;\mathrm{AVERAGE}\;\mathrm{AREA}\times 20\times 1.5},$$

where Ws is the weight of the LVN standard, in mg; Wu is the assay percentage of the LVN standard; and 1.5 refers to the theoretical assay result of one tablet.

**FIGURE 2:**
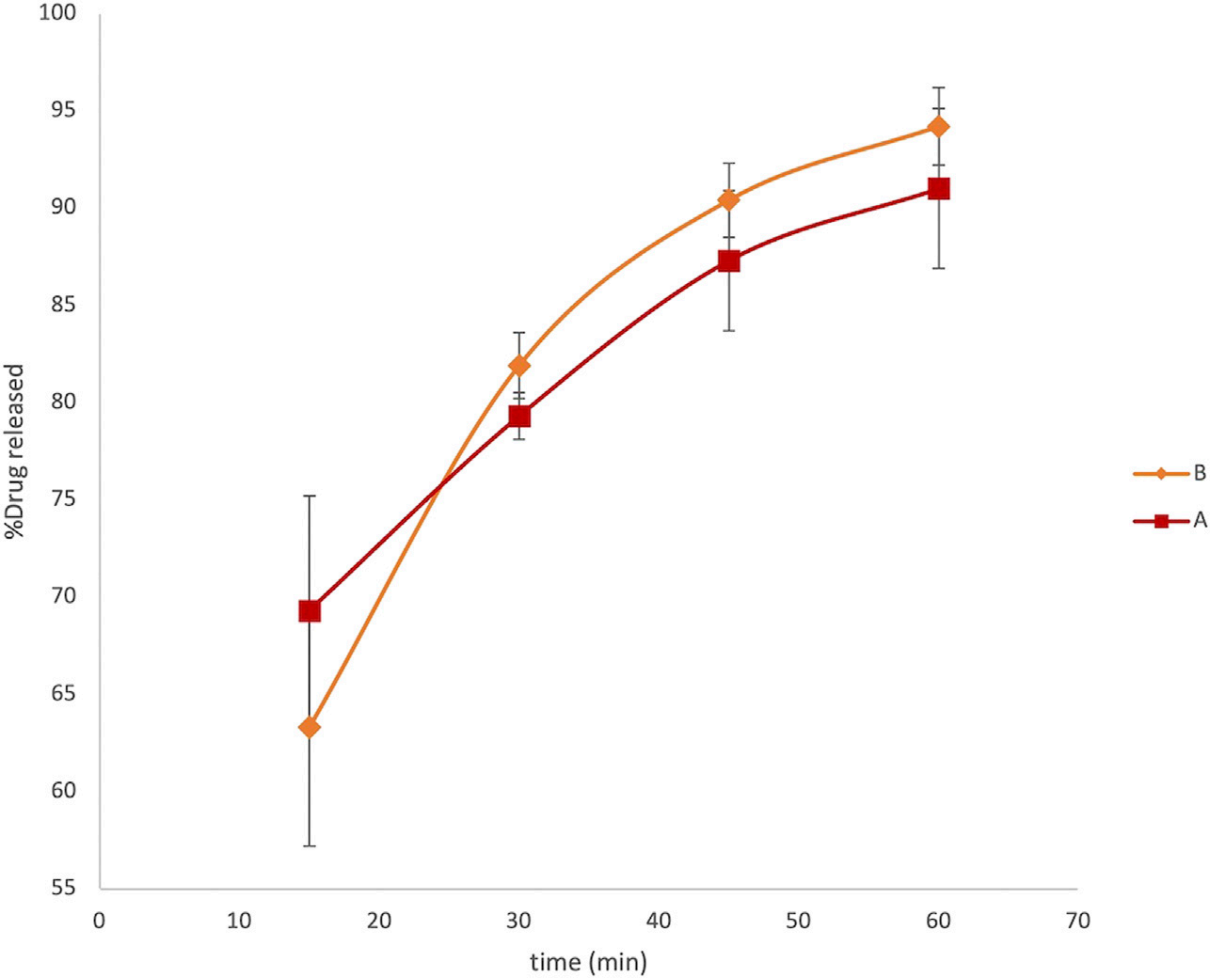
LVN delivery profiles obtained by HPLC analysis. The graph shows the percentage of drug released vs time in minutes. Data are the mean of six replicates. RSD% ranged from 1.2 to 6.1%.

The graph reported in Figure 2 describes a release profile very close to both the formulations. The original and generic formulations showed an extremely similar pattern of dissolution, both reaching the maximum released concentration equal to 1.36 ± 0.05 μg ml^−1^ and 1.41 ± 0.02 μg ml^−1^ in 1 h for formulations A and B, respectively. This analysis did not reveal any substantial evidence of different behavior between the generic and brand-name LVN formulations failing to explain the unsuccessful results obtained in *in vivo* BE studies. Moreover, we also calculate the fit factors f1 (similarity factor) and f2 (difference factor) applying the following equations:

$$f1=\left\{\left[\overset{n}{\underset{t=1}{\sum }}\left|Rt-Tt\right|\right]\text{|}\left[\overset{n}{\underset{t=1}{\sum }}Rt\right]\right\}x100,$$

$$f2=\;50\cdot log\left\{{\left[1+\frac{1}{n}\overset{n}{\underset{t=1}{\sum }}{\left(Rt-Tt\right)}^{2}\right]}^{-0,5}x100\right\},$$

where Rt is the percentage of the dissolved product at time point t for the reference formulation (formulation B), Tt is the percentage of the dissolved product for the test batch, and n is the number of time points. The comparison of this value gives the opportunity to easily calculate and compare pairs of dissolution profiles. The values obtained were equal to 0.88 and 85.67 for f1 and f2, respectively, indicating a very similar releasing profile for the two drugs (Anderson et al., 1998).

#### UV Spectroscopy Analysis

UV spectroscopy was used to detect and quantify LVN release kinetics during the dissolution test as an alternative to HPLC analysis. The LVN standard solution UV spectrum, examined between 210 and 350 nm, shows one absorption band with a maximum at 245 nm. Therefore, the linearity of the method was assessed by a calibration curve obtained by using standard solutions of LVN at different concentrations (0.375–6 μg/ml). A linear calibration graph was obtained by plotting A_245_ against the LVN standard corresponding concentrations. The obtained equation was *y* = 0.045 ± 0.004× + 0.009 ± 0.003 (*n* = 6; correlation coefficient 0.9952). The LoD and LoQ were found to be 5.6 and 56 ng/ml. The UV method was found to be selective, since the excipients did not interfere at the LVN absorption maximum wavelength. The selectivity was proved by comparing the absorption spectra registered for LVN standard solution, LVN solution obtained after the dissolution test, and the one obtained after the dissolution of tablets without API.

**FIGURE 3:**
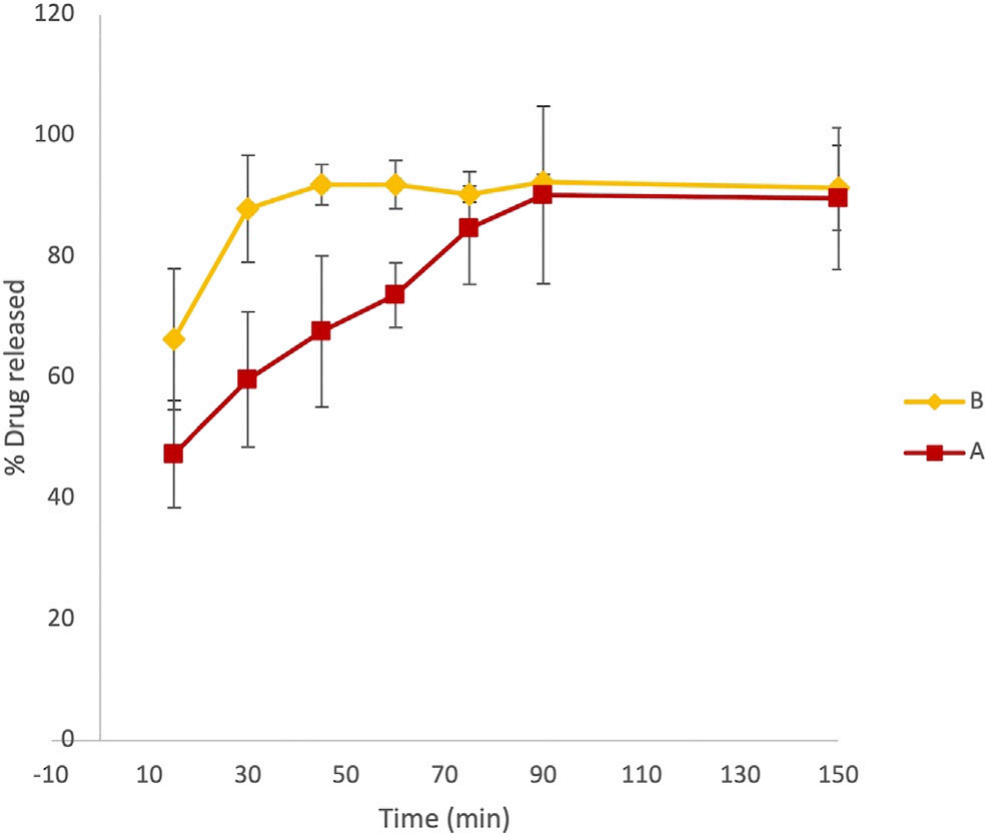
Overlaid kinetic plots showing the percentage of LVN released by formulations A and B in time-course experiments. Data are the mean of six replicates. RSD% ranged from 1 to 15%. The graph clearly shows a difference in the dissolution trend for the two tablets.

At fixed time intervals, ranging from 15 to 150 min, aliquots of 3 ml samples were withdrawn from paddles and replaced with an equal volume of dissolution media. The absorbance of LVN in different samples was registered at 245 nm. Therefore, the application of the zero-order UV analyses to the LVN analytical solution was performed, which allowed for a selective determination of LVN in the dissolution experiments. The concentration of LVN expressed as μg/ml, for each sample, was calculated interpolating the absorbance values in the calibration curve. The results were then expressed as percentage of the LVN theoretical maximum concentration released from the tablet content, namely, 1.5 μg/ml. The time course of LVN release during the dissolution test, for both formulation A and formulation B, expressed as % of released drugs, is reported in Figure 3. Data points are the mean of three independent experiments, each repeated three times. RSD% was in the range from 1 to 15%. Unlike the results obtained by HPLC analysis, the graph reported in Figure 3 shows that the kinetic profile for the release of LVN from both the tablets is quite different. Indeed, the fit factors obtained were found to be equal to 16.18 and 63 for f1 and f2, respectively, indicating a different releasing profile for the two drugs.

This difference is already significant in the first 30 min (Figure 3). Indeed, the LVN concentration values of 0.89 and 1.32 μg/ml were calculated for formulations A and B, respectively, by applying the equation obtained from the calibration curve and transforming the absorbance values registered for the two spectra of the solutions after 30 min dissolution. After 30 min, the percentage of LVN released by formulations A and B was 60 and 88%, respectively. The maximum amount of levonorgestrel released from formulation B is quickly obtained after 45 min following the results obtained by HPLC analysis (Figure 2). In contrast, at this time, the percentage of LVN released by formulation A is still 67%, lower than that detected by HPLC analysis. Afterward, at longer times, there is still a substantial difference between the amount of LVN released from the two tablets: the rate of LVN released from formulation A is 20% lower than the one from formulation B. In fact, for formulation A, the maximum amount of LVN released is stabilized after 90 min, twice as long as formulation B’s. This result is in agreement with the high BA characterizing the studied drug and with the reported *T*_max_ equal to 1.4 h (Devoto et al., 2005). Despite that the equipment used to carry out this assay might present limitations in its ability to replicate the dynamic process of the oral forms in the complex GI luminal environment, the evaluation/prediction of bioequivalence of solid oral preparations can be considered reliable. Moreover, since the active compound studied belongs to class I of the BCS, it is reasonable to consider the obtained results as robust and reliable. Since the two formulations have the same qualitative and quantitative composition, the differences noticed in the release of LVN can be ascribed to the physicochemical difference, such as granulometry superficial areas, in excipient/drug physicochemical status. LVN from formulation A might establish different stronger non-covalent interactions with excipients, causing a decreased solubility. The application of UV spectroscopy, as the detection method, made it possible to detect and quantify LVN solubility, excluding the possibility of false-positive results. Despite that the dissolution method was the same, analyzing the two LVN different samples by UV spectroscopy, the theoretical maximum drug concentration was not attained until 90 min, demonstrating the formation of insoluble LVN excipient aggregates. Instead, the application of HPLC analysis to the same study did not reveal any kinetics difference in the amount of LVN released by the two pharmaceutical products during the dissolution experiment. In this case, the percentage of LVN released in 60 min for both the formulations resulted to be the same and very close to 100%. This result can be related to the presence of an organic modifier in the mobile phase, which can solubilize drug/excipient aggregates caused by non-covalent interactions with excipients, hypothetically due to different solid-phase states between same excipients (Paus et al., 2015; Zarmpi et al., 2017). Other differences may arise in the morphology of more stable and less soluble LVN crystals used for the two formulations.

#### PAMPA Test Validation

Five standard compounds of known permeability were used to validate the permeability assay. Atenolol, carbamazepine, coumarin, norfloxacin, and ranitidine hydrochloride are indeed characterized by different oral permeability ranging from low to high values. The concentrations of drug solutions, before incubation, and of those collected from the acceptor wells, after incubation time, were determined by UV measurements in the wavelength range from 210 to 450 nm. The permeability coefficient (Pe) of each drug, in centimeter per second, was then calculated by applying the following formula (Avdeef et al., 2007):

$$\begin{array}{l} Pe=\frac{Vd\cdot Vr}{\left(Vd+Vr\right)\cdot S\cdot t}\text{In}\frac{100\cdot Vd}{100\cdot \text{Vd}-\text{\%T}\left(\text{Vd}+\text{Vr}\right)},\\ \%T=\frac{Vr\cdot Ar}{Ad_{0}\cdot Vd}100, \end{array}$$

where $V_{d}$ and $V_{r}$ are the volume of the donor and the receptor solutions (0.18 cm^3^), S is the membrane area (0.266 cm^2^), t is the time of incubation expressed in seconds, Ar is the absorbance of the receptor plate after the experiment, and Ad_0_ is the initial absorbance in the donor compartment (Alex, 2012).

**FIGURE 4:**
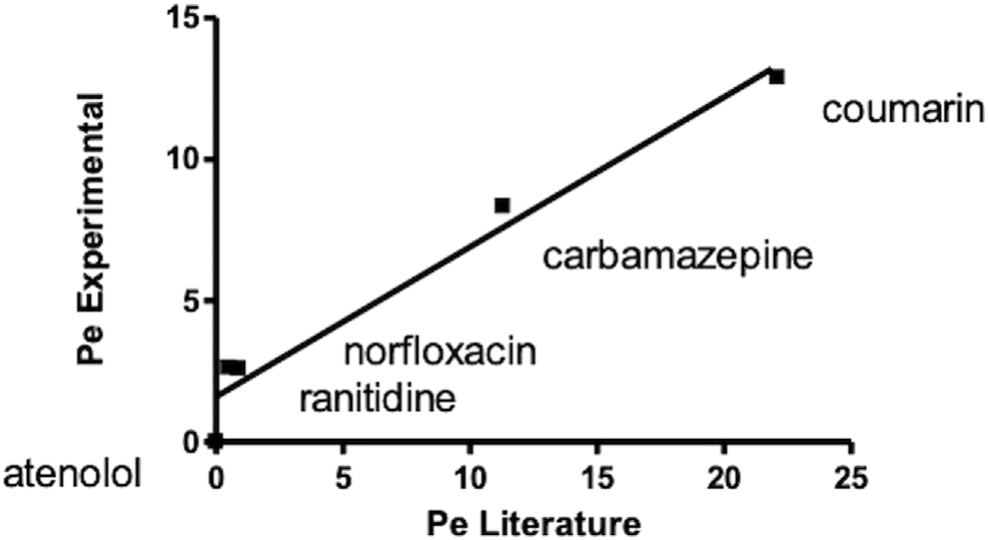
Correlation graph for experimental and theoretical Pe of standard compounds obtained carrying out a PAMPA test for oral absorption assessment.

A correlation graph for experimental and theoretical Pe obtained for quality control compounds revealed a good correlation between the reported Pe (Zhu et al., 2002) and experimental ones (*R* ^2^ > 0.95) (Figure 4).

#### LVN Stability

As reported before (*PAMPA Test Validation*), the Pe value of substances determined by the PAMPA test is calculated taking into account the absorbance of analyte solutions in the receptor plate, after the experiment, and the initial absorbance of the analyte solutions in the donor compartment. The LVN solutions obtained by performing the dissolution test, as previously described, were not suitable for the PAMPA test since they showed A_245_ values corresponding to too low concentrations (<1.35 μg/ml) to get adequate analytical sensitivity. In order to determine the LVN Pe by the PAMPA test, we optimized the concentration for LVN solution suitable for the assay. The tested solution has indeed to be stable and has to ensure the assay sensitivity. Moreover, since the determination of Pe by the PAMPA requires long incubation time, ranging from few hours to more than 12 h, the stability of LVN in assay conditions was investigated. The absorbance of LVN at different concentrations ranging from 6 to 30 μg/ml in 0.1% SDS 0.1N HCl was monitored over 6 days. The obtained results showed a substantial decrease in the absorbance value, for the 30 μg/ml solution, during the first 24 h. At concentrations equal to 15 and 6 μg/ml, the absorbance values registered over days did not change, demonstrating LVN stability.

For these results, the PAMPA test was applied to 15 μg/ml LVN standard solution since it represents the highest concentration at which LVN solutions are stable.

#### PAMPA Test Applied to LVN Tablets

##### Dissolution Assay in 100 ml Volumetric Flasks

**FIGURE 5:**
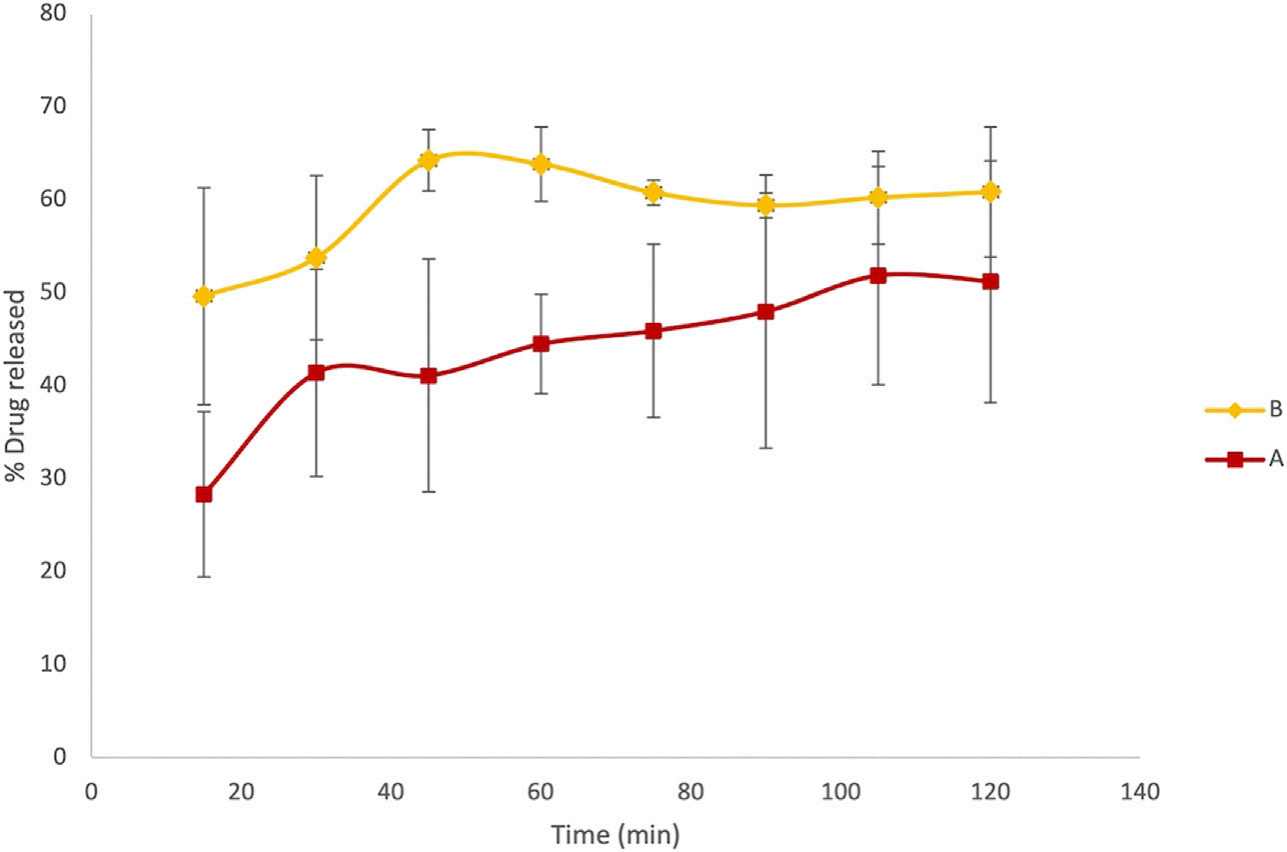
Results referring to the dissolution test adjusted to obtain more concentrated LVN solutions, suitable for the PAMPA test. Overlaid kinetic plots show the percentage of the time-course LVN release from generic **(A)** and brand-name tablets **(B)**.

Since the maximum concentration of LVN solution obtained by applying the paddle apparatus for the dissolution test was 1.35 ± 0.18 μg/ml and 1.35 ± 0.03 μg/ml for formulations A and B, respectively, we reproduced the dissolution test in a 100 ml flask in order to obtain solutions with concentrations close to 15 μg/ml. Indeed, one of the most common experimental issues in carrying out a permeability assay is to provide sufficient concentration levels of the drug in order to easily evaluate the permeability value (Lakeram et al., 2008; Buckley et al., 2012). Thanks to the previously shown advantages, the use of UV spectroscopy as the detection method was applied also in this experiment. The kinetic profile obtained for formulations A and B was very similar to that achieved by the dissolution test carried out according to European Pharmacopoeia 9.0, described in paragraph 2.1.2. The results, expressed as percentage of LVN released over time, are quite similar to those obtained by using the dissolution test as reported in Figure 5, showing *T*_max_ at 45′ and 105′ for formulation B and formulation A, respectively.

##### PAMPA

We applied this method to understand the differences in BE that emerged in the *in vivo* studies of two LVN oral formulations. Since the aim of this work was to offer a dissolution/permeation model to apply for the BE study, we adopted the LVN solutions obtained from the dissolution test, performed as reported in *Dissolution Assay in 100 ml Volumetric Flasks*, in order to investigate the effect of drug/excipient interactions also on permeability in a time-course experiment. The use of solutions derived from the dissolution test allowed to keep LVN in the dissolved state, thus avoiding the formation of supramolecular assemblies that could have altered the results by precipitation on the layer of the membranes (Flaten et al., 2008; Fischer et al., 2011a). Moreover, the application of solutions obtained as described in *Dissolution Assay in 100 ml Volumetric Flasks* allowed us to reach an optimal concentration level of the drug in the receiver compartment, overcoming analytical difficulties that may occur since the concentrations obtained may be lower than the limit of detection of the most common method of analysis such as HPLC (Liu et al., 2003). However, the choice and the pH of the medium used to carry out the assay are an essential step. They have to fulfill the criterion of analyte solubility and do not have to alter the test conditions. The membrane integrity indeed has to be preserved.

**FIGURE 6:**
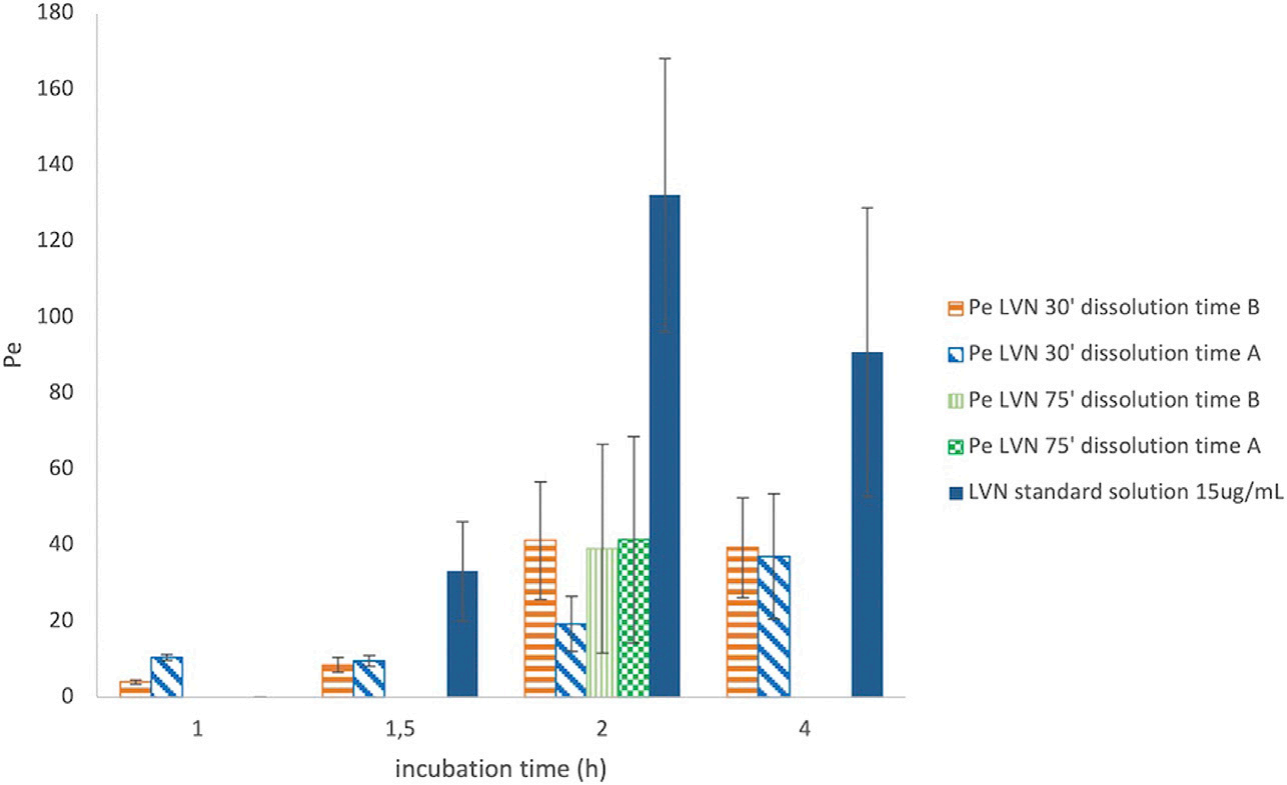
Graph reporting the Pe values for LVN released from both formulations A and B after 30′ and 75′ dissolution time. The Pe values are plotted against different incubation time in hours. Data are the mean of at least four replicates. RSD% ranged from 7.70 to 70.23%.

After PAMPA test validation carried out by using standard compounds of known Pe coefficient, the same system has been adopted for the characterization of LVN permeability released by both the 1.5 mg formulations A and B. Solutions containing LVN released by both the tablets at 30 and 75 min, during the dissolution test, carried out as reported in *Dissolution Assay in 100 ml Volumetric Flasks*, were used in the PAMPA test, in order to highlight the permeability when the extent of LVN released by the tablet is different. As shown before, 30′ and 75′ represent the times at which the maximum and minimum differences, for the amount of LVN released by formulations A and B, were obtained during the dissolution test. The permeability assay was then carried out at different incubation times, ranging from 30 min to 4 h. In parallel, a 15 μg/ml LVN standard solution was applied to the same procedure. The Pe values obtained for LVN released by both the tablets and LVN 15 μg/ml standard solution, at increasing times, are reported in Figure 6. In this graph, the Pe values are plotted against the PAMPA experiments’ incubation times. The two formulation solutions, taken at 30 min from the release kinetic experiments, showed the most different Pe values. A Pe value of 19.33 × 10^−6^ cm/s was obtained for LVN from formulation A, significantly lower than and almost half of the formulation B Pe value of 41.26 × 10^−6^ cm/s. After 2 h, the results were very similar when the LVN solution was taken from the release at 75 min. The maximum Pe values of 39.14 × 10^−6^ and 41.49 × 10^−6^ cm/s are reached for LVN released by formulations B and A, respectively, presumably because at this time the maximum extent of release was reached by both the tablets. For the LVN standard solution, the maximum Pe value (132.14 × 10^–6^ cm/s) was reached after 2 h incubation time. This result could be ascribed to the absence of excipients that could interfere with the permeation of the drug.

The results are the mean value of four different experiments and are reported in Table 1. The RSD% value ranges from 8 to 70%. The results show a significant difference between formulations A and B at 30′ dissolution time, 2 h incubation in the PAMPA, in agreement with the dissolution experiment UV data (Figures 3, 5). In contrast, the highest RSD% values were found for the experiments carried out on solutions derived from the 75′ dissolution test and by applying 2 h incubation time. Despite the high RSD% value, the difference in Pe mean values obtained for LVN derived from both the tablets can be considered not statistically significant (*p* > 0.05). This variability could be ascribed to the decrease in membrane stability probably due to the higher incubation time.

**TABLE 1:** Mean values and RSD% of the LVN Pe. The results were obtained by applying the equation reported in PAMPA test validation paragraph taking into account the different incubation times. The results refer to both the experiments carried out after 30′ and 75′ dissolution time. The reported values are the mean of four independent experiments.

Incubation time (h)	Formulation B 30′ dissolution time		Formulation A 30′ dissolution time		Formulation B 75′ dissolution time		Formulation A 75′ dissolution time	
	Pe mean value	RSD%	Pe mean value	RSD%	Pe mean value	RSD%	Pe mean value	RSD%
1	4.04	13.78	10.54	7.70				
1.5	8.54	12.89	9.53	15.00				
2	41.26	37.53	19.33	37.72	39.14	70.23	41.49	65.42
4	39.99	33.33	37.06	44.33				

With that in mind, an incubation time of 2 h was found to be suitable for determining the LVN Pe value. The PAMPA data obtained after 2 h incubation time clearly show that LVN released from formulation B was able to reach the highest Pe value in half an hour. The obtained results underline the substantial difference, already highlighted in the dissolution test, in the amount of LVN released from the two tablets over time (almost half rate for formulation A vs formulation B). Therefore, PAMPA test results gave a clear picture of the absorption profile of LVN released from the two oral formulations and demonstrated that the LVN/excipient insoluble aggregates cannot pass through the GI tract.

## Conclusion

With the intention of developing a new approach for the characterization of the *in vitro* BA profile of oral formulations, we focused on the case study of LVN. The determination of the dissolution and permeability profiles of two oral formulations, a generic and a brand-name one, was carried out to set up a reliable *in vitro* model to resolve or predict problems in *in vivo* BE studies.

By using a solvent simulating the intestinal fluid, a dissolution test was carried out in order to compare the LVN solubility and kinetics of release from the two oral formulations A and B. Interestingly, the UV spectroscopy direct determination of the LVN solutions obtained from the dissolution test in a time-course experiment was able to show different LVN kinetic releases in the two formulations, with a much slower dissolution profile for the generic formulation. In fact, it was found out that the presence of acetonitrile in the mobile phase of the HPLC system altered LVN solubility, increasing the dissolution of insoluble aggregates. As a consequence, by using the HPLC analysis, statistically equal LVN kinetics of release were determined for the two formulations, predicting a wrong equal behavior in *in vivo* experiments, which conversely failed to demonstrate *in vivo* BE.

Meanwhile, the optimization of the PAMPA for drug tablets allowed us to determine LVN permeability as an indication of passive oral absorption of LVN released by both formulations. This parameter was found to be statistically different for LVN generic and brand-name formulations, obtained after the 30′ dissolution test (*p* < 0.05).

Thus, the combination of solubility and PAMPA methods demonstrated to be predictive of *in vivo* BE. Indeed, the capability of the *in vitro* model to predict the unmet BE studies among the two studied oral formulations was demonstrated. The obtained results explained and justified the unsuccessful results of the *in vivo* BE study. The studied case provided important insights into confirming that the new approach combining solubility and permeability studies can be useful in revealing crucial issues in the prediction of *in vivo* BE. Hence, during the formulation process, the two combined approaches can be recommended to analyze the drug performances resulting from dissimilar ingredient/drug interactions in generic formulations.

## Data Availability

The raw data supporting the conclusions of this article will be made available by the authors, without undue reservation.
