# Supplementary figures and images for: Combined Methodologies for Determining In Vitro Bioavailability of Drugs and Prediction of In Vivo Bioequivalence From Pharmaceutical Oral Formulations
Source: Front Chem. 2021 Nov 3;9:741876. doi: 10.3389/fchem.2021.741876 (PMC8597939; doi:10.3389/fchem.2021.741876)

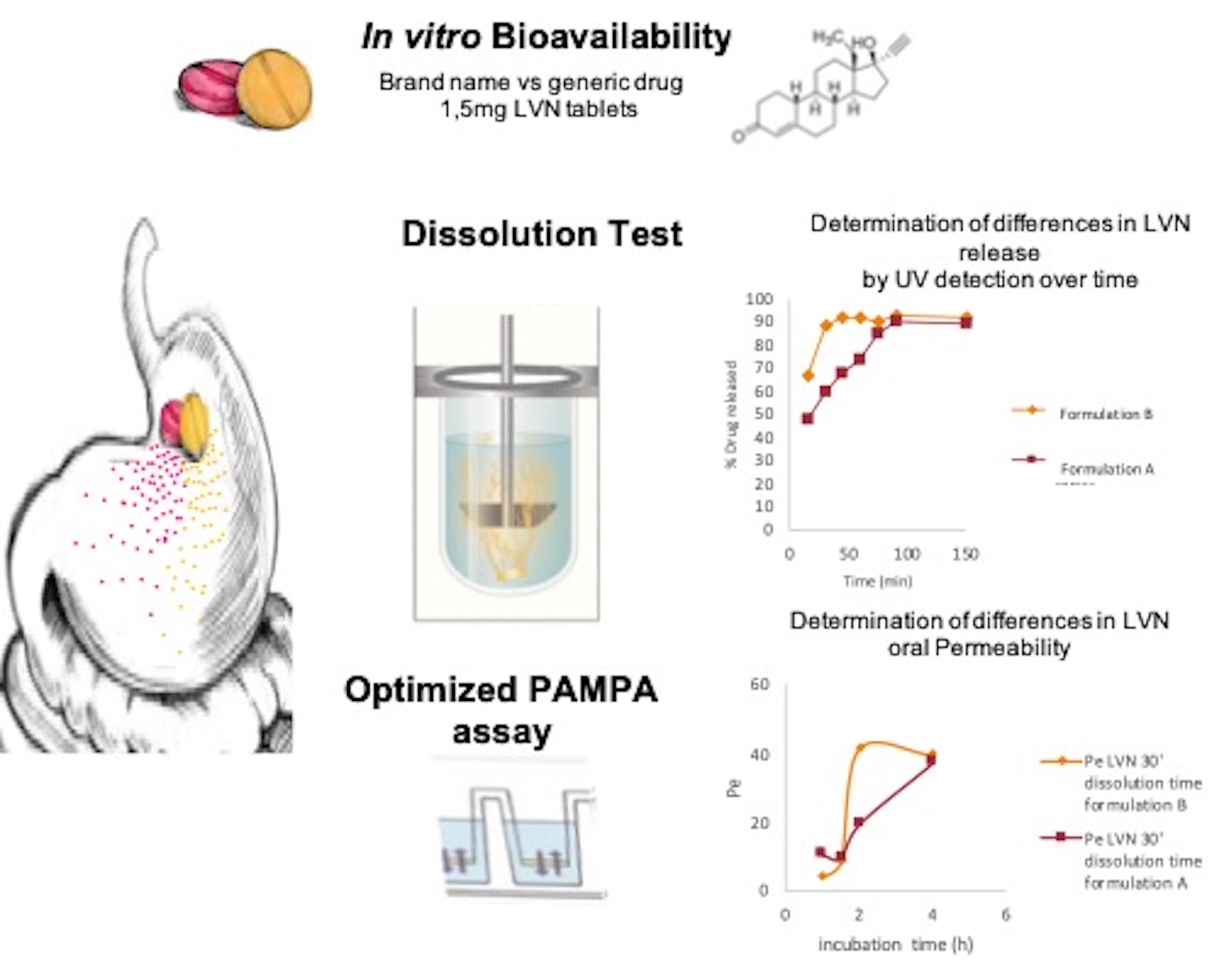

Supplement: Supplementary file 1 [file Image1.jpg]
